# Epidemiology and Risk Factors in Non-infectious Uveitis: A Systematic Review

**DOI:** 10.3389/fmed.2021.695904

**Published:** 2021-09-10

**Authors:** Katherine A. Joltikov, Ann-Marie Lobo-Chan

**Affiliations:** Department of Ophthalmology and Visual Sciences, Illinois Eye and Ear Infirmary, University of Illinois at Chicago College of Medicine, Chicago, IL, United States

**Keywords:** non-infectious uveitis, epidemiology, risk factors, systematic review, incidence, prevalence

## Abstract

**Purpose:** Non-infectious uveitis is a leading cause of vision loss in the developed world. The purpose of this systematic review is to investigate the epidemiology and risk factors of non-infectious uveitis over the last 50 years.

**Methods:** A systematic literature search of Pubmed/MEDLINE database was performed in the 50-year period from January 1971 to January 2021, according to the PRISMA guidelines. Studies that assessed the epidemiology and risk factors for non-infectious uveitis were included.

**Results:** Few epidemiologic studies focus specifically on non-infectious uveitis. In the Unites States, the estimated prevalence of non-infectious uveitis is 121/100,000. The incidence and prevalence varies considerably worldwide. Females and the working age group (20–50 years) appear to be the most affected. Smoking and vitamin D deficiency are the biggest risk factors for non-infectious uveitis, while pregnancy appears to be protective. Additional risk factors include presence of other autoimmune diseases (thyroid disease, diabetes, celiac), pre-eclampsia/eclampsia, psychological stress, and certain medications (bisphosphonates, immune checkpoint inhibitors, female hormone therapy, and etanercept).

**Discussion:** Our systematic review summarizes the incidence and prevalence of non-infectious uveitis and associated modifiable and non-modifiable risk factors.

## Introduction

Uveitis refers to inflammation of the uveal tissues of the eye, including the iris, ciliary body, and choroid. Other intraocular structures can also be involved in uveitis, including the sclera (termed scleritis), retina, retinal blood vessels, and the optic nerve. Uveitis can be associated with significant visual morbidity, with over one-third of patients with uveitis having visual impairment (1). In the developed world, it is the 5th or 6th leading cause of blindness, accounting for about 10–15% of all cases of blindness (2, 3). Unlike other ocular diseases, such as glaucoma or age-related macular degeneration, which generally affect elderly populations, uveitis can occur in all age groups and often affects young adults (4, 5).

Uveitis is categorized as infectious or non-infectious. Non-infectious uveitis can occur with systemic autoimmune disease and autoimmune diseases localized to the eye. Etiologies of non-infectious uveitis include HLA-B27 associated anterior uveitis, Fuchs uveitis syndrome, sarcoidosis, Vogt-Koyanagi-Harada (VKH), sympathetic ophthalmia, birdshot chorioretinopathy, multifocal choroiditis, serpiginous choroiditis, and Behçet disease. Non-infectious uveitis represents the majority of uveitis cases (67–90%) in the developed world (6–8). Most previous epidemiologic studies combine infectious and non-infectious etiologies together. Although risk factors for infectious and non-infectious uveitis may overlap, the causes of the inflammation are inherently different and require different management approaches. For this review we chose to include only non-infectious causes of uveitis, to avoid obscuring any epidemiologic data and risk factors specific to non-infectious causes.

Multiple factors influence the development of non-infectious uveitis, including age, sex, race and ethnicity, environmental and social factors, genetics, systemic conditions, and certain medications. The purpose of this systematic review is to investigate the epidemiology and risk factors of non-infectious uveitis in adults over the last 50 years.

## Materials and Methods

A systematic literature search, data extraction, statistical analyses and assessment of the quality of evidence were performed according to a pre-specified protocol using the PRISMA guidelines (9).

### Search Strategy

For this systematic review, the authors conducted an electronic database search of Pubmed/MEDLINE using a combination of keywords related to uveitis (non-infectious/non-infectious uveitis, ocular inflammation, HLA-B27 uveitis, sarcoid uveitis, VKH, and Behçet uveitis) and epidemiology (prevalence, incidence, population, risk factors, and survey). The search period was from January 1971 to January 2021. The articles deemed relevant were cross-referenced for additional manuscripts which were not directly found through the above search.

### Eligibility Criteria

Studies published between January 1971 and January 2021 were included in this systematic review if they met the following inclusion criteria: (1) population-based cross-sectional or cohort studies or large case series [with at least 20 patients] (2) uveitis clearly defined as non-infectious (3) articles written in English, (4) studies performed in humans, (5) full-text available. Exclusion criteria included: (1) self-reported diagnosis of uveitis, (2) studies in persons <18 years of age.

### Data Collection and Extraction

Two reviewers (KAJ and AMLC) conducted data extraction based on the inclusion and exclusion criteria above. Data extracted included the first author's name, year of publication, study location, sample size, data collection methods, prevalence and incidence of non-infectious uveitis, anatomic location of inflammation (anterior, intermediate, posterior, panuveitis), duration/chronicity of inflammatory process (acute, recurrent, chronic) and demographic and other factors including age, sex, race and ethnicity, environmental and social factors, genetics, systemic conditions, and medications.

### Synthesis of Evidence

The same information was extracted, when available, from all included studies. A meta-analysis of all included studies was not able to be conducted because of study population heterogeneity and differences in the methodology of the included studies.

## Results

### Description of Studies

**Figure 1:**
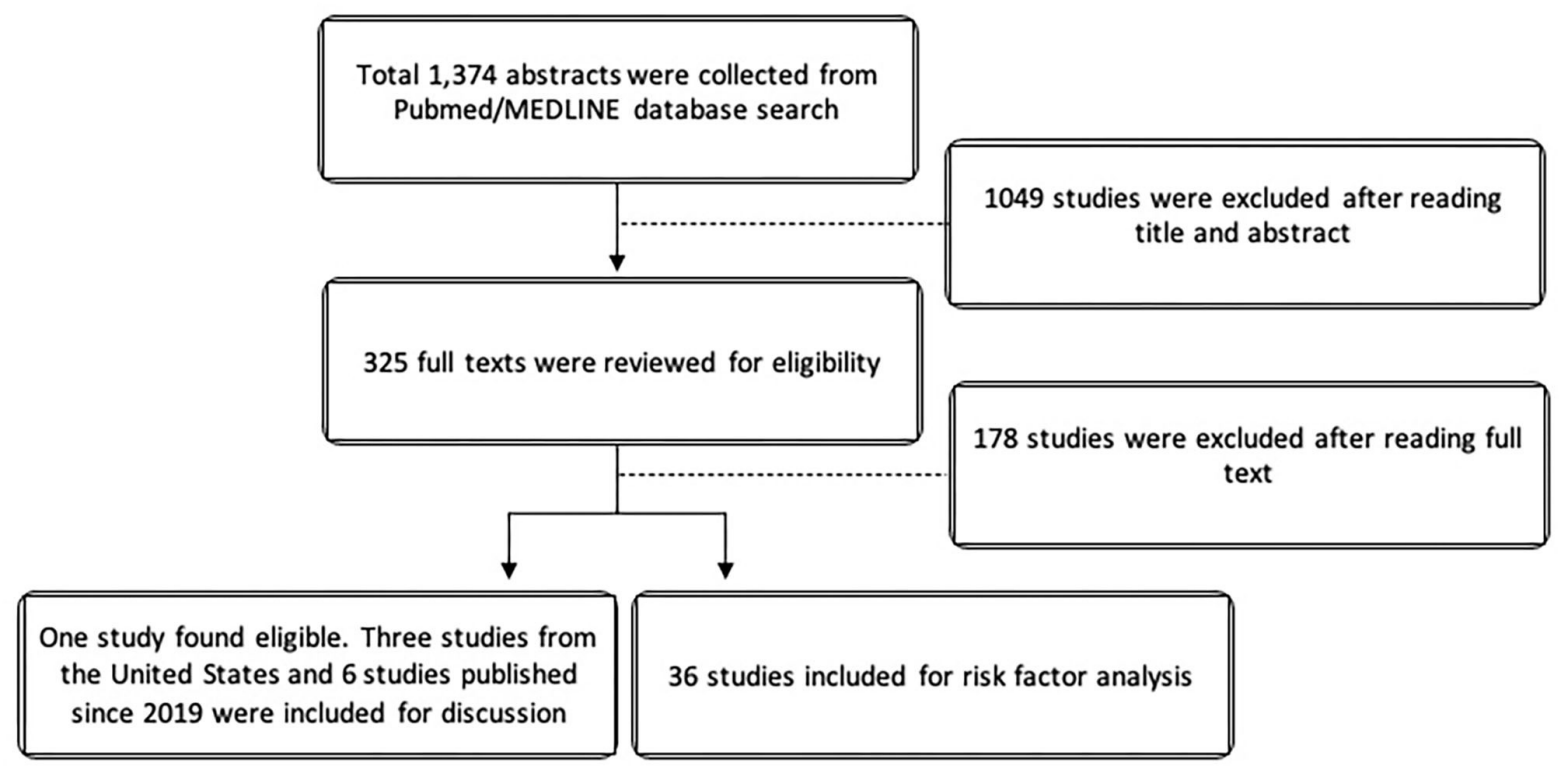
PRISMA flow diagram for literature selection.

The search yielded 1,374 studies from Pubmed/MEDLINE databases. After cross-referencing for additional relevant studies, there was a total of 325 studies that were reviewed. For the epidemiologic analysis, only one study specifically evaluated non-infectious uveitis in terms of prevalence. Due to the paucity of epidemiologic data on non-infectious uveitis, we elected to also described the epidemiology of uveitis in the United Stated using the 3 large population-based studies from the United States in the last 50 years. Since Hsu et al. (10), Tsirouki et al. (5), and García-Aparicio et al. (11) have already extensively reviewed the incidence and prevalence of uveitis worldwide, we elected to minimize references and describe only the most recent international studies published since January 2019. For risk factor analysis, 36 studies were included. Figure 1 illustrates the PRISMA flow diagram for literature selection.

### Epidemiology of Non-infectious Uveitis

#### Incidence and Prevalence in the United States

In the United States, only one study specifically met our search criteria for the prevalence of adult non-infectious uveitis. Thorne et al. used a medical claims database of almost 4 million patients throughout the United States and showed that the prevalence of adult non-infectious uveitis was 121 per 100,000 persons. Given the methodology of the study, Thorne et al. was not able to assess incidence of non-infectious uveitis (7).

Over the last 50 years, there have been 3 large population-based epidemiologic studies of uveitis in the United States, and they included infectious causes of uveitis, such as herpetic, histoplasmosis, toxoplasmosis, tuberculosis, bartonella neuroretinitis, and HIV/AIDS (6, 12, 13). Two studies came from the Kaiser Permanente Health system: one in Hawaii (Pacific Ocular Inflammation Study) and one in Northern California (Northern California Epidemiology of Uveitis Study). The Pacific Ocular Inflammation Study found an annual incidence of uveitis of 24.9, while the Northern California study found an incidence of 52.4 cases of uveitis per 100,000 person-years (6, 12). The third study, from the Pacific Northwest Veterans Administration (VA), found the annual incidence of uveitis to be 25.6 per 100,000 person years (13). There may be several reasons for large variability in uveitis incidence between these studies, including demographics of the study populations.

While data on the incidence of uveitis is helpful in understanding the rate of new cases in a particular time period, prevalence of uveitis may be more meaningful since many patients with uveitis may develop a chronic and/or recurrent course. The population study from Northern California reported a prevalence of 114.5 per 100,000 adults (6), which is similar to the prevalence reported by Thorne et al. of 121 per 100,000 persons (7). However, comparing the two directly is not possible as the Northern California study included cases of infectious uveitis. In the Veterans Administration study, the prevalence was lower at 69 per 100,000 persons, likely due to a primarily male population compared to other studies (13). Likewise, the population studied in the Pacific Ocular Inflammation study out of the Kaiser Health system in Hawaii contributed to lower prevalence of uveitis of 58 per 100,000, likely because Pacific Islanders have a lower prevalence of uveitis compared to other racial/ethnic groups studied in other population-based studies (12). Table 1 summarizes the findings of these 4 epidemiologic studies from the United States.

**Table 1:** Large population-based epidemiologic studies of uveitis in the United States.

**Country**	**Year**	**Method**	**Sample size**	**Age**	**Incidence (per 100,000 person-years)**	**Prevalence (per 100,000 persons)**	**% Non-infectious**	**Sex**	**Location of Uveitis**	**Chronicity**	**Limitations**
USA (7)	2012	Medical claims database	3,994,054 total. 4,827 uveitis cases	Included all ages. Prevalence increased with age	NA	121	All non-infectious	Females with higher prevalence than males (*p* < 0.05)	81% anterior		• Possible misclassification bias from using ICD-9 codes • Retrospective nature of the study did not allow to assess incidence of NIU
USA (Hawaii) (12)	2006–2007	Population based cohort	217,061 total. 872 uveitis cases	Included all ages. Incidence increased with age	24.9	58.0		Females with higher prevalence than males (*p =* 0.008)	72% anterior	43% acute, 26% recurrent, 25% chronic	• Selection bias for Pacific Islanders population • Included: Herpetic, histoplasmosis, toxoplasmosis, tuberculosis, bartonella neuroretinitis, HIV/AIDS
USA (Northern California) (6)	1998–1999	Population based cohort	731,898 total. 844 uveitis cases	Included all ages. Prevalence increased with age	52.4	115.3		Incidence similar in males and females (*P =* 0.29). Prevalence higher in females (*p* < 0.001)			• Included: Herpetic, histoplasmosis, toxoplasmosis, tuberculosis, Toxocara, bartonella neuroretinitis, HIV/AIDS
USA (VA in Pacific Northwest) (13)	2004	Population based cohort	152,267 total. 126 uveitis cases	Age >25. Trend toward higher prevalence in younger age group (25–44)	25.6	69	80% non-infectious	Only 8.0 % females in study population. No significant difference between males and females	83.3% anterior		• Selection bias, primarily male population • Included: Herpetic, histoplasmosis, toxoplasmosis, tuberculosis, Toxocara, bartonella neuroretinitis, HIV/AIDS

#### Incidence and Prevalence Worldwide

Worldwide the incidence and prevalence rates of non-infectious uveitis varies widely. Like in the United States, literature specific to only non-infectious uveitis is scarce. We were not able to identify any international studies that met our search criteria, since many studies include both infectious and non-infectious, and idiopathic etiologies. In most cases, idiopathic etiology was non-infectious (10). In 2019 Hsu et al. described the epidemiology of non-infectious uveitis in the Asia Pacific region (10). The prevalence of uveitis ranged from 152 per 100,000 persons in China, to 173 per 100,000 persons in South Korea (14), to 194 per 100,000 persons in Taiwan (15). The prevalence from studies in India ranged from 317 per 100,000 (16) to 730 per 100,000 (17). Importantly, these large population based studies did not distinguish between infectious and non-infectious etiologies. In 2018, Tsirouki et al. described the heterogeneity of the incidence and prevalence of uveitis worldwide (5). In 2021, García-Aparicio et al. published a systematic review and meta-analysis of the prevalence and incidence of uveitis, including studies published up until January 2019 (11). To avoid reviewing already extensively reviewed manuscripts, Table 2 describes the most recent epidemiologic studies published since January 2019, not previously reviewed by Hsu et al. (10), Tsirouki et al. (5), and García-Aparicio et al. (11). Our goal of reviewing these studies, it to update the knowledge on the incidence and prevalence of uveitis since 2019. Table 2 shows that the prevalence of uveitis varies from 12.4 per 100,000 persons in Portugal (19) to 580 per 100,000 persons in Thailand (22). Once again, these recent studies did not specifically focus on non-infectious uveitis, although most of the cases were of non-infectious etiology (18–23).

**Table 2:** Summary of large population-based epidemiologic studies worldwide published since Jan 2019^*^.

**Country**	**Year**	**Method**	**Sample size**	**Incidence** **(per 100,000 person-years)**	**Prevalence** **(per 100,000 persons)**	**% Non-infectious**	**Age**	**Sex**	**Location of Uveitis**	**Chronicity**	**Limitations**
Australia (18)	2014–2015	Retrospective cohort	1,236 cases	21.5	36.3	86.6% non-infectious	Mean age of diagnosis 46.2	No significant difference between males and females	75% anterior	NA	• Authors acknowledge that unknown number of uveitis cases within Melbourne were not included due to external management (thus study likely underestimates true prevalence in urban Australia)
Portugal (19)	2012–2017	Retrospective cohort	545 cases	3.9	12.4	45.5% non-infectious; 28.4% idiopathic	Mean age of diagnosis 47.8	No significant difference between males and females	47.5% anterior	NA	• Likely biased toward more severe diseases (referral center bias) • Selection bias for northern Portuguese population
Spain (20)	2016–2017	Retrospective cohort	529,855 sample size. 358 cases	NA	67.6	84% non-infectious	Mean age of diagnosis 47.0	No significant difference between males and females	83.2% anterior	NA	• Cross-sectional retrospective nature of the study limits evaluation of incidence of uveitis
Sweden (21)	2013–2017	Retrospective cohort	2,483 cases	108 (2013–2017)	700 (2013–2017)	86% idiopathic; 9.2% non-infectious		No significant difference between males and females	93% anterior	NA	• Possible misclassification bias from using ICD-10 codes • Selection bias
Thailand (22)	2013–2018	Retrospective cohort	101,203 sample size. 586 cases with uveitis	NA	580	44% non-infectious; 36% idiopathic	Mean age of diagnosis was 46.3	No significant difference between males and females	50% anterior uveitis	57.7% acute	• Single center • Tertiary military care center more biased toward more severe cases • Selection bias for Thai population
China (23)	2008–2018	Retrospective cohort	15,373 cases of uveitis	NA	NA	32.1% non-infectious, 53.4% idiopathic	Mean age of onset 35.4. Prevalence increased with age	Prevalence of systemic diseases in uveitis was 37.0% for males and 23.6% for females (p>0.001)	62.8% panuveitis	NA	• Likely biased toward more severe diseases (referral center bias) • Selection bias for Chinese uveitis patients • No follow-up information • Lack of information on visual outcomes

* *Only included studies not previously reviewed by Hsu et al. (10), Tsirouki et al. (5), and García-Aparicio et al. (11)*.

#### Location of Uveitis and Chronicity

Uveitis is classified according to anatomic location in the eye: anterior, intermediate, posterior, or panuveitis. Location of uveitis is important as it can portend visual compromise and development of ocular complications, such as cataract, macular edema, and glaucoma. Additionally, uveitis can be classified based on duration and chronicity of disease, including acute onset uveitis, recurrent uveitis, and chronic uveitis (24).

As can be seen from Tables 1, 2, anterior location was the most common location of non-infectious uveitis, representing 47.5 to 93% of cases. After anterior uveitis, panuveitis and posterior uveitis have similar frequency of about 20% of cases of uveitis and intermediate uveitis is the least common form of uveitis at ~10–15% of cases (5–7, 10, 12–15, 19, 21, 22, 25, 26). From the study by Throne et al. it was estimated that 10% of anterior uveitis can be classified as severe, requiring more advanced therapies than topical corticosteroids (7). Interestingly, McCannel et al. demonstrated that there were significantly more cases of unilateral acute anterior uveitis seen in a community-based practice (90.6%) compared to those seen in a tertiary care hospital (60.6%), which saw more cases of chronic anterior uveitis (27). This study suggested that most studies on uveitis patients from tertiary referral centers were subject to referral bias as these centers treated the most severe cases (27).

In terms of chronicity, the Pacific Ocular Inflammation study demonstrated that most cases of uveitis (43%) were acute. It is unclear how many of these are non-infectious. From the recent Thailand study (22), acute uveitis represented more than half of cases (57.7%), which included 44% non-infectious and 36% idiopathic cases. Additional studies are needed to elucidate further epidemiologic trends in chronicity of non-infectious uveitis.

### Demographics of Non-infectious Uveitis

#### Age

Uveitis can occur in all age groups. Many studies have previously demonstrated a high incidence of uveitis in the working age groups (20–50 years) (5, 10, 18, 19, 21, 25). Interestingly, Thorne et al. showed that non-infectious uveitis increases with age (7). Similarly, the Pacific Northwest VA study, which had 80% non-infectious cases, showed increased incidence with increasing age, however it did have limited data on younger age groups (13). These studies suggest that while uveitis can affect individuals throughout their lifetime, there may be a higher burden of disease in the elderly than originally suspected from earlier studies. Possible reasons for increased incidence of disease in older adults include higher likelihood of prior ocular surgery which can contribute to inflammation (28) and increased incidence of underlying autoimmune disease with age (29).

#### Sex

In the United States, there is a higher frequency of female adult patients with uveitis than male adult patients (6, 7, 12). Both the Pacific Ocular Inflammation Study and the Northern California Epidemiology of Uveitis Study demonstrated a higher prevalence of uveitis in female compared to male patients, and the Northern California Epidemiology of Uveitis Study demonstrated a higher rate of uveitis in females among all age groups (6, 12). In the study by Thorne et al, 56.8% of adult patients with non-infectious uveitis were female (7). Since the primary cause of non-infectious uveitis is related to autoimmune disease and autoimmune diseases are more prevalent in adult females, it is reasonable to anticipate that autoimmune uveitis may be more frequent in adult female patients. Sex hormones, including estrogen, likely contribute to the differing immune response and susceptibility to development of autoimmune diseases, including uveitis, in women (30). Many studies worldwide found that males and females are equally affected by uveitis, however these studies included both infectious and non-infectious causes of uveitis (5, 10, 18–22).

#### Race/Ethnicity

Among the many subtypes of uveitis, including uveitis associated with systemic diseases, there can be certain racial predilections. For example, Behcet disease, which can be associated with severe anterior uveitis and occlusive retinal vasculitis, is more commonly seen in racial/ethnic groups along the Silk Road, including the Middle East and Asia (5, 10, 31). Vogt Koyanagi Harada syndrome, which is associated with a bilateral granulomatous panuveitis, is more commonly seen in races/ethnicities with more skin pigmentation, including Asian, Native American, and Hispanic individuals (10, 32, 33). In the Pacific Ocular Inflammation Study, there was a higher incidence of uveitis in the Caucasian/white and black racial groups compared to Pacific Islanders (12).

### Association With Systemic Disease

The most common known cause of non-infectious uveitis in the developed world is HLA-B27 associated uveitis (5). The overall prevalence of HLA-B27 in the United States is 6.1%, with non-Hispanic whites having the highest prevalence of all other races/ethnicities at 7.5% (34). Prevalence of HLA-B27 varies widely, with 15.9% reported in Norway (35) and <1% reported in Japan (36). It is estimated that 30–80% of patients with seronegative spondyloarthropathies are HLA-B27 positive (37, 38). Patients may be tested for HLA typing after presenting with a recurrent acute anterior uveitis in order to screen for risk of seronegative spondyloarthropathies. In a large nationwide cohort study in Korea, the incidence rate ratio for development of ankylosing spondylitis (AS) increased with every episode of recurrent acute anterior uveitis, with a rate of 277.3 (95% CI 171.6–423.8) for more than 2 episodes of uveitis (39).

Sarcoidosis represents another systemic disease that can be associated with the development of eye disease in 30–60% of patients, including 20–30% of cases with non-infectious uveitis (40). Sarcoidosis is more common in African American and Asian patients, but can be seen in all populations (5, 10). Uveitis in sarcoidosis can be acute or chronic and can precede any systemic or extraocular manifestations; in one recent study, one-third of patients with ocular sarcoidosis developed symptomatic systemic disease within 16 months of when the uveitis started (41). The manifestations of ocular sarcoidosis may differ based on race, with black patients more likely to develop chronic anterior uveitis and white patients more likely to develop posterior uveitis (40).

Behçet disease is a multisystem inflammatory disease that is diagnosed based on clinical criteria including ocular inflammatory disease and recurrent oral and genital ulcers. The prevalence of Behçet disease has significant geographic variation with pooled global prevalence of 10.3 per 100,000 inhabitants, but with prevalence as high as 119 per 100,000 in Turkey to 2.1 per 100,000 for Northern Europe (31). Behçet disease can be associated with severe uveitis, with panuveitis being the most commonly observed location for uveitis (42). Occlusive retinal vasculitis is another common manifestation. A recent study by Sota et al. compared 64 juvenile-onset and 332 adult-onset Behçet disease patients, and showed that those with juvenile-onset Behçet disease (first manifestation of disease before age 16) have a lower prevalence of uveitis than adult-onset Behcet disease (42). Male patients may have higher risk for severe ocular involvement than female patients (43).

### Risk Factors Associated With Non-infectious Uveitis

As non-infectious uveitis represents a heterogeneous group of diseases, there are many risk factors which have been associated with the development or progression of uveitis. As previously mentioned, certain demographic variables, including age, female gender, and certain races/ethnicities may be more predisposed to developing uveitis. However, there are a number of external risk factors that may contribute to development of disease. Table 3 summarizes the potential factors that influence the development of non-infectious uveitis as described over the last 50 years, including smoking, vitamin D levels, pregnancy, autoimmune disease, and certain medications. Each study included was identified according to a pre-specified protocol using the PRISMA guidelines, and limitations of each study are listed in Table 3.

**Table 3:** Risk Factors for Non-infectious Uveitis.

**Risk Factor**	**Country**	**Year**	**Method**	**Sample size**	**Findings (odds ratio = OR, hazard ratio = HR, relative risk = RR, confidence internal = CI)**	**Study Limitations**
Smoking	USA (Hawaii) (44)	2006–2007	Retrospective Cohort	80 uveitis, 522 general controls, 528 ophthalmology controls	Active smokers had increased risk of uveitis compared to never smokers in general population (OR 2.10, 95% CI 1.10–3.99) Active smokers had increased risk of uveitis compared to ophthalmology controls (OR of 2.96, 95% CI 1.52–5.77)	• Information on duration and quantity of cigarette smoking not available • Authors acknowledge possible misclassification of non-infectious uveitis
Smoking	USA (Wilmer) (45)	1984–2006	Cross-sectional	208 uveitis (only intermediate uveitis)	Active smokers had increased risk of cystoid macular edema (CME) compared to never smokers (OR 3.90, 95% CI: 1.43–10.66) Former smokers had increased risk of CME compared to never smokers (OR 1.97, 95% CI: 0.99–3.94)	• Single center study • Cross-sectional study design did not allow to establish causal relationship • Patient self-reporting smoking data (reporting bias)
Smoking	USA (Procter) (46)	2002–2009	Retrospective case-control	222 uveitis, 564 ophthalmology controls	Active smokers had increased risk of uveitis compared to never smokers (OR 2.2, 95% CI 1.6–2.8)	• No information on onset of uveitis in relation to onset of smoking • No information on quantity of smoking or type of smoke exposure • Reporting bias
Smoking	Germany (47)	2011	Cross-sectional	350 uveitis	Active smokers had increased risk of uveitis compared to never smokers (OR 1.8, 95% CI 1.2–2.9)	• Single center study • Reporting bias • Cross-sectional study design did not allow to establish causal relationship
Smoking	Portugal (48)	2019	Cross-sectional	40 spondylarthritis related uveitis	Lifetime (ever) smoking was associated with uveitis (OR 2.54, 95% Cl 1.01–6.42)	• Single center study • Cross-sectional study design did not allow to establish causal relationship
Smoking	Turkey (49)	2008–2013	Retrospective cohort	202 Behçet uveitis	No statistically significant differences found between smokers and non-smokers	• Single center study • No information on quantity of cigarette smoking, duration of smoking, or previous smoking
Smoking	United Kingdom (50)	2012–2017	Prospective cohort	484 spondylarthritis related uveitis	Current smokers had lower incidence of uveitis compared to non-smokers (OR) 0.7 (95% Cl 0.5–0.9)	• Selection bias (studying smoking status within population of axSpA patients)
Vitamin D	USA (Mass Eye and Ear) (51)	2008–2015	Retrospective case-control	100 anterior uveitis	Low vitamin D level associated with uveitis (OR 2.53, 95% Cl 1.42–4.51)	• Single center • Cross-sectional study design did not allow to establish causal relationship
Vitamin D	USA (52)	2005–2016	Retrospective case-control	333 uveitis, 103 scleritis, and 329 controls	Low vitamin D level associated with uveitis (OR 1.92, 95% CL 1.36–2.72)	• Selection bias for controls with available vitamin D data • Retrospective nature of study cannot exclude unknown confounders
Vitamin D	USA (53)	2000–2016	Retrospective case-control	558 uveitis and 2,790 controls	Normal vitamin D levels were protective for uveitis (OR 0.79, 95% Cl 0.62–0.99), especially in black patients (OR 0.49; 95% CI 0.30–0.80)	• No assessment of supplemental use of vitamin D (possible miscategorization bias) • No information on confounders i.e., smoking status
Vitamin D	Turkey (54)	2017	Prospective case-control	20 uveitis and 20 controls	Serum vitamin D levels in acute anterior uveitis (5.75 ± 4.50 ng/mL) were significantly lower than the control group (12.96 ± 5.89 ng/mL)	• Small sample size
Vitamin D	Romania (55)	2014–2015	Prospective case-control	34 ankylosing spondylitis, 18 controls	Serum vitamin D levels in acute anterior uveitis (16.50 ng/mL) significantly lower than for patients with ankylosing spondylitis without uveitis (26.40 ng/mL) [*p* < 0.05]	• Small sample size
Vitamin D	Australia (56)	2017	Prospective case-control	151 uveitis and 594 controls	Vitamin D level in the active uveitis was significantly higher than in the inactive group (*P* < 0.001). Reduced odds of active uveitis compared with inactive uveitis as serum vitamin D levels increased (OR 0.98; 95% Cl 0.96–0.99)	• Cases and controls not matched demographically
Pregnancy	USA (57)	1983–2003	Retrospective cohort	32 pregnant uveitis; 32 non-pregnant uveitis; and 32 healthy non-pregnant controls	Rate of flare-ups during pregnancy was lower than during non-pregnant periods (1.0 vs. 2.4 per year; *p* < 0.001) and lower than in control group (3.1 per year; *p* < 0.001)	• Retrospective nature of study
Pregnancy	Australia (58)	1985–−2011	Retrospective case series	47 pregnant uveitis	Rate of flare-ups during pregnancy was lower than pre-pregnancy (*p* < 0.001) Rates of flare-up began to decrease in the 2nd trimester. Within 6 months postpartum, flare-up rates not significantly different from pre-pregnancy level	• Retrospective nature of study and incomplete data on follow-up
Pregnancy	Saudi Arabia (59)	1983–2001	Observational case-series	50 pregnant uveitis	A flare-up in uveitis activity occurred within the first 4 months of pregnancy in 49 of 76 cases (64%) and later in pregnancy in 17 cases (22%). A flare-up within 6 months of delivery occurred in 38 of 59 cases (64%)	• Retrospective nature of study and incomplete data on follow-up • Bias against using systemic medications during pregnancy
Pregnancy-eclampsia	Taiwan (60)	1997–2012	Population based retrospective cohort	2,229 pre-eclampsia/eclampsia (PEE), 99,953 controls	Incidence of uveitis was higher among post-delivery women with PEE than without PEE, (incidence rate ratio 2.96, 95% Cl 1.48–5.90). Risk of uveitis was higher in PEE group than in the non-PEE group (HR 2.96, 95% CI 1.48–5.92)	• Potential misclassification bias as diagnosis of PEE and NIU dependent on ICD-9 codes • Did not differentiate between nulliparous and multiparous • Systemic medications were not evaluated
Thyroid	USA (Hawaii) (61)	2006–2007	Retrospective case control	224 patients with uveitis, 896 controls	Patients with thyroid disease had a 1.7-fold higher odds of having uveitis (95% CI, 0.97–2.9)	• Possible misclassification bias using ICD-9 codes • Possible misclassification of non-infectious uveitis
Thyroid	Taiwan (62)	2000–2012	Retrospective cohort	21,396 patients with thyroid disease	Patients with thyroid disease had higher risk of uveitis (HR 1.54, 95% Cl 1.36–1.75)	• Possible misclassification bias using ICD-9 codes • Selection bias (Taiwanese population)
Diabetes	United Kingdom (63)	2010–2015	Retrospective cohort	938,440 total, 48,584 with diabetes	Uveitis associated with type 1 diabetes (OR 2.01, 95% CI 1.18–3.41) and type 2 diabetes (OR 1.23, 95% CI 1.05–1.44). Poor glycemic control associated with uveitis ( OR 4.72, 95% CI 2.58–8.65)	• Retrospective nature of study cannot exclude unknown confounders and cannot confirm causal relationship
Celiac Disease	Sweden (64)	1969–2008	Retrospective cohort	29,044 patients with celiac disease	Celiac disease associated with uveitis (HR) 1.32 (95% CI 1.10 to 1.58)	• Lack of data on dietary adherence • Retrospective nature of study cannot exclude unknown confounders and cannot confirm causal relationship
Psychological stress	USA (65)	2017–2018	Cross-sectional case-control	120 patients with uveitis, 40 controls	Having uveitis was associated with a 4.3 point increase on the perceived stress scale (95% Cl 1.8–6.9) compared to controls	• Cross-sectional study design did not allow to establish causal relationship • Questionnaire recall bias
Psychological stress	Austria (66)	2006–2008	Cross-sectional survey	171 patients with HLA-B27	Patients with HLA-B27 had 4.3 point increase on Beck Depression Inventory scale (*p* < 0.001) compared to controls	• Response rate of 45% • Questionnaire recall bias • Cross-sectional study design did not allow to establish causal relationship
Bisphosphonates	Canada (67)	2000–2007	Retrospective cohort study	10,827 new bisphosphonate users, and 923,320 controls	Increased risk of uveitis (RR 1.45, 95% CI 1.25–1.68) and scleritis (RR 1.51, 95% CI 1.34–1.68) in first time bisphosphonate users compared with nonusers	• Authors acknowledge possible misclassification bias of uveitis
Bisphosphonates	USA (68)	2005–2006	Retrospective cohort	35,252 new bisphosphonate users, 3,736 new uveitis, among 5.7 million veterans	No increase in risk of uveitis associated with new bisphosphonate prescriptions (RR = 1.23, 95% CI 0.85–1.79)	• Use of de-identified medical data and possible misclassification bias
Bisphosphonates	New Zealand (69)	2012	Randomized, double masked, placebo controlled clinical trial	2,001 women	Increased incidence of uveitis associated with intravenous zoledronate infusions (incidence of 0.8%)	• Possible under-reporting of ocular side effects • Only 1 patient was re-challenged with the drug
Fluoroquinolones (FQ)	Canada (70)	2001–2011	Case-control	13,313 cases, 133,130 controls	First-time users of moxifloxacin had increased risk for uveitis (RR 2.98, 95% CI, 1.80–4.94). First-time users of ciprofloxacin had increased risk for uveitis (RR 1.96, 95% CI, 1.56–2.47). Levofloxacin not associated with uveitis (RR 1.26, 95% CI 0.90–1.77)	• Cohort limited to older men • Possible misclassification bias as results are based on drug prescriptions rather than drug use
Antibiotics	Canada (71)	2000–2007	Case-control	3,383 cases of uveitis and 33,830 controls	FQ associated with uveitis (RR 3.53, 95% CI 2.84–4.39). Macrolides associated with uveitis (RR 2.37, 95% CI 1.76–3.20). Beta-lactams associated with uveitis (RR 1.36, 95% CI 1.01–1.84)	• Possible misclassification bias as results are based on drug prescriptions rather than drug use
Fluoroquinolones	USA (72)	2000–2013	Retrospective cohort	843,854 on FQ, 35,43,797 on beta-lactam	No association between uveitis and FQ use (HR 0.96, 95% CI, 0.82–1.13). Association between FQ use and the risk for uveitis-associated systemic illnesses (HR range, 1.46–1.96; 95% CI, 1.42–2.07)	• Possible misclassification bias as results are based on drug prescriptions rather than drug use
Immune checkpoint inhibitors (CPI)	USA (73)	2010–2019	Retrospective cohort, insurance database	8,678 CPI exposed and 76,153 on non-CPI chemotherapy	CPI exposure increased risk for uveitis compared to the non-CPI chemotherapy (HR = 2.09; 95% CI 1.36–3.22)	• Single US insurer data • Use of de-identified medical data and possible misclassification bias
Immune checkpoint inhibitors	USA (74)	2010–2020	Retrospective chart review	996 patients on CPI	Uveitis occurred in 4 patients out of 996 on CPI (incidence 0.4%)	• Data does not account for different dosing regimens • Possible misclassification bias using ICD-10 codes
Female hormone replacement (FHT)	USA (75)	2000–2016	Retrospective cohort, insurance database	217,653 women exposed to FHT and 928,408 women not unexposed to FHT	FHT cohort was more likely to develop uveitis (HR, 1.21, 95% CI, 1.04–1.41) women on FHT aged ≥45 years more likely to develop uveitis (HR, 1.23, 95% CI, 1.03–1.47)	• Use of de-identified medical data and possible misclassification bias
Etanercept	USA (76)	1998–2006	Retrospective cohort, database review	43 cases of uveitis with etanercept, 14 cases with infliximab, and 2 cases with adalimumab	Etanercept was associated with increased risk of uveitis compared to infliximab (*P* < 0001, OR 5.375)	• Possible misclassification bias since 50% of cases had unknown underlying systemic diagnosis • Lack of information on duration of exposure to medications
Etanercept	Global (77)	2010	Metanalysis of 8 clinical trials	1,709 subjects included across 8 clinical trials for ankylosing spondylitis	Etanercept associated with lower uveitis rates than placebo (8.6 per 100 subjects vs. 19.3 per 100 subjects, respectively) (*p =* 0.03). Etanercept was associated with similar uveitis rates to sulfasalazine (10.7 per 100 subjects vs. 14.7 per 100 subjects, respectively) (*p =* 0.49)	• No information on prior history of uveitis • Lack of information on duration of exposure to medications
Etanercept	France (78)	2000–2014	Observational, retrospective study	429 subjects included; 203 received monoclonal antibodies and 226 received etanercept	Uveitis risk lower with Etanercept than monoclonal antibody, but difference was not statistically significant (OR 0.94, 95% CI 0.35-2.54, *P =* 0.90)	• Single center study • Monitoring bias • Recovery of missing data by phone calls (recall bias)

#### Smoking

Among cross-sectional, case-control, and population-based studies conducted on the effect of smoking on uveitis, there is general agreement among studies that smoking increases the risk for development of ocular inflammation (44–48). From the Pacific Ocular Inflammation Study, smoking was associated with a 2 times greater odds of developing new onset non-infectious uveitis compared to patients who never smoked (44). In a large case-control study, smoking was found to increase the odds of having ocular inflammation in all anatomic types of uveitis, with higher odds in posterior and panuveitis compared to anterior uveitis; both current and past smokers had a 2 times higher odds of developing uveitis compared to those who had never smoked (46). A cross-sectional study of 350 non-infectious uveitis patients showed that in addition to active uveitis, smoking was also associated with younger age of uveitis activity, more frequent topical corticosteroid dosing, and a dose-dependent higher odds of macular edema and cataract (47). Smoking was associated with a 4-fold increased risk of cystoid macular edema in a dose-dependent manner in patients with intermediate uveitis; macular edema is a leading cause of vision loss in uveitis (45). It is hypothesized that smoking causes endothelial cell dysfunction with resultant increased leakage of retinal blood vessels and development of macular edema; while other factors associated with aging may also effect retinal vasculature, smoking was still associated with uveitis and macular edema when adjusting for age and other systemic diseases (45).

Interestingly, in a large prospective study from the United Kingdom, smoking was found to be protective against uveitis in a population of patients with spondyloarthropathies. As the authors point out, selection bias may be contributing to this finding (50). In another retrospective cohort study from Turkey, Bilgin et al. found no statistically significant association with Behçet uveitis among smokers and non-smokers. Limitations of this study include its single center patient population, and lack of information about smoking quantity, duration of smoking, and history of prior smoking (49).

#### Vitamin D

Vitamin D plays a role in immune regulation and vitamin D deficiency has been associated with a number of autoimmune diseases, including multiple sclerosis, inflammatory bowel disease, and rheumatoid arthritis. Low vitamin D levels have also been found in a number of studies on patients with uveitis, including patients with systemic diseases associated with uveitis (51–56).

In a small case-control study examining patients with anterior uveitis at a single institution, low vitamin D levels were seen in a significantly greater number of patients with uveitis than in control patients (51). A larger study from the same institution demonstrated that for every 1 nanogram/ml increase in vitamin D level, there was a 5% lower odds of having uveitis; this study also demonstrated association of low vitamin D levels with anterior uveitis, as well as panuveitis and scleritis (52). In a large case-control study using a national health insurer database, normal vitamin D levels were associated with a 21% lower odds of developing uveitis compared to low vitamin D levels; these results were even more pronounced in black patients with a 51% lower odds of having uveitis with normal vitamin D levels compared to low vitamin D levels (53).

A study on patients with idiopathic or HLA-B27 associated acute anterior uveitis demonstrated that these patients had significantly lower vitamin D levels compared to controls (54). In a study examining vitamin D levels and other immune markers in patients with AS, low vitamin D levels were associated with acute anterior uveitis and uveitis flares compared to AS patients without anterior uveitis (55).

One recent large case-control study demonstrated a difference in vitamin D levels among patients with active and inactive uveitis; prior studies had not differentiated between disease activity, just disease onset in relation to vitamin D levels. Patients with active uveitis had lower levels of vitamin D compared to those with inactive disease and population-based controls; increased sunlight exposure among those with vitamin D deficiency was associated with disease inactivity (56). These results provide some background for further studies into vitamin D supplementation through sunlight exposure or supplementation as a way of preventing inflammatory disease flares, although a large randomized control trial would be needed to confirm these findings.

#### Pregnancy

As previously discussed, non-infectious uveitis is more prevalent in female patients and can affect women of any age, including those of child-bearing potential. Because of the role of sex hormones in the development of autoimmune disease, the course of uveitis may change in pregnancy and during the post-partum period when there are significant changes in levels of estrogen and progesterone. During pregnancy, elevated levels of estrogen and progesterone are associated with a decline in Th1 type immunity in order for the immune system to tolerate the semi-allogeneic fetus (79). This decline in Th1 type immunity, with decline in cytokines such as tumor necrosis factor alpha, interleukin-12 and interferon gamma, also contributes to improvement in Th1 mediated autoimmune conditions such as multiple sclerosis and rheumatoid arthritis. Conversely, there may be potential worsening of Th2 mediated and antibody mediated conditions such as systemic lupus erythematosus. Many forms of uveitis are also thought to be Th1 mediated and several small series have indicated that the course of uveitis improves in pregnancy (57–59). In one study, uveitis activity levels were found to decline during pregnancy (second and third trimesters) compared to pre-pregnancy, with flare up rates of 0.540 and 1.188 per person year, respectively (58). The rate of uveitis flares was not significantly different from pre-pregnancy levels by 6 months post-partum. These findings supported a prior study that found that uveitis flare ups peaked in the first trimester of pregnancy and then declined in the second and third trimesters with a similar rate of flare ups pre-pregnancy and post-partum (57).

A recent population based cohort study in Taiwan examining the relationship between pre-eclampsia/eclampsia and non-infectious uveitis demonstrated a three times higher incidence rate of non-infectious uveitis in women who had pre-eclampsia/eclampsia compared to age, urbanization, and income-matched controls (60). None of these women with pre-eclampsia/eclampsia who developed non-infectious uveitis had underlying autoimmune or thyroid disease. It has been postulated that during pre-eclampsia/eclampsia, there may be a shift from Th2 back to Th1 mediated immunity, which may be why pre-eclampsia/eclampsia represent a potential risk factor for development of non-infectious uveitis (60).

#### Thyroid Disease

The association of thyroid disease and non-infectious uveitis has been studied (61, 62). From the Pacific Ocular Inflammation study, patients with thyroid disease had a 1.7 higher odds of developing non-infectious uveitis compared to controls without thyroid disease (61). More recently, a large cohort study from Taiwan found a 1.54 higher risk of developing non-infectious uveitis in patients with thyroid disease. Males without thyroid disease had a lower risk of developing uveitis than females (62). Both thyroid disease and uveitis have an autoimmune component, which may be why thyroid disease is a potential risk factor for non-infectious uveitis.

#### Diabetes

Both diabetes and uveitis can cause vision loss through various mechanisms and disruption of the blood-ocular barrier. One large cohort study in the United Kingdom examined the relationship between diabetes, glycemic control and uveitis using a large primary care database of over 1 million patients; they found that acute anterior uveitis occurred more commonly in patients with diabetes than without diabetes (63). Poor glycemic control, defined as HbA1c >11.3, and proliferative retinopathy were also strongly associated with acute uveitis. This study was limited by using diagnosis codes from a primary care database which do not specify the etiology of uveitis. It may also have ascertainment bias since patients with diabetes are more likely to have routine eye exams than healthy controls. The finding of type 1 diabetes having a stronger association with uveitis may reflect the fact that both result from immune system dysfunction and are considered autoimmune diseases.

#### Celiac Disease

As an immune-mediated disease of the small intestine associated with ingestion of gluten, celiac disease has also been associated with the development of other autoimmune diseases, including uveitis (80). In a large nationwide cohort study in Sweden, individuals with biopsy-proven celiac disease had a higher risk of development of uveitis (hazard ratio 1.32, 95% CI 1.10–1.58) compared to controls (64). However, this study did not specify the etiology of uveitis. Nonetheless, in those individuals with celiac disease and uveitis, both diseases may respond to strict adherence to a gluten-free diet. The microbiome and disruptions in the microbiome have also been hypothesized to play a role in autoimmune diseases, including celiac disease, inflammatory bowel disease, and uveitis (80).

#### Psychological Stress

Psychological stress can alter immune function, and chronic stress is associated with an attenuated immune response over time (81, 82). In a cross-sectional survey study from Austria, it was found that patients with HLA-B27 associated uveitis had significantly higher scores on the Beck Depression Inventory scale, a standardized survey for depression, compared to healthy controls. Almost 60 percent of patients with HLA-B27 associated uveitis in this study reported life events and psychological distress as potential triggers for uveitis flares (66). In a recent case-control study, Berlinberg et al. found that patients with non-infectious uveitis self-reported a 4.3-point increased score on a 10-item perceived stress scale compared to patients without uveitis. In multivariate analysis, female sex and history of depression were most strongly associated with an increased perceived stress score. However, there was no difference in the perceived stress score between active and controlled uveitis patients. Measures of diurnal salivary cortisol levels were not significantly different between patients with and without uveitis (65). It remains unclear if psychological stress is a risk factor of development of non-infectious uveitis, but it appears to play a role regardless of disease activity.

#### Medication-Induced Uveitis

Various medications have been associated with the development of uveitis. Most cases of drug-induced uveitis are single case reports or small case series rather than larger cohort or case-control studies. Some reports may also come from post-marketing surveillance of drugs, but may lack specific details on the course of uveitis.

##### Bisphosphonates

The bisphosphonates are the most commonly used class of medications to prevent the development of osteoporosis. Both oral and intravenous bisphosphonates have been associated with an increased risk for development of uveitis and scleritis, possibly due to the release of inflammatory cytokines such as tumor necrosis factor alpha and interleukin-6. One large retrospective cohort study from British Columbia found 1.45 increased risk for the development of uveitis in first-time oral bisphosphonate users compared to those not on bisphosphonates. However, they did not specifically identify the uveitis as non-infectious (67). Another study from a veterans population found that there was no significant difference in rates of uveitis among those prescribed bisphosphonates and the general veteran population (relative risk: 1.23, 95% CI 0.85–1.79) and that overall rates for uveitis were low (68). Another study using data from a randomized controlled trial of women receiving intravenous zoledronate compared to placebo found that the incidence of uveitis was 0.8% and occurred only in the zoledronate arm of the study (69). Unlike the other 2 studies which were performed using diagnosis codes and ran the risk of misclassification bias, the study on intravenous zoledronate by Patel et al. included only uveitis cases confirmed by an ophthalmologist (69).

##### Antibiotics

Certain antimicrobial medications have been associated with development of uveitis. Fluoroquinolones have been associated with bilateral acute transillumination defects, iris atrophy and pigment dispersion with anterior uveitis. In a case-control study conducted using a cohort of men from a large health claims database in British Columbia, first time users of moxifloxacin had an increased risk for uveitis (rate ratio: 2.98, 95% CI 1.80–4.94) compared to nonusers of fluoroquinolone antibiotics (70). The rate of uveitis was higher with moxifloxacin compared to other fluoroquinolones such as ciprofloxacin and levofloxacin (70). A nested case-control study done in the same population of patients in British Columbia examined the use of fluoroquinolone antibiotics as well as macrolide and beta-lactam antibiotics in new cases of uveitis compared to age-matched controls (71). An increased risk for uveitis was found among fluoroquinolone users, but also among those on macrolide and beta-lactam antibiotics which have not been associated with uveitis; the authors concluded that there was no concrete evidence of an association between fluoroquinolone use and uveitis. In fact, it is possible the uveitis was post-infectious or part of the systemic prodrome.

A large cohort study using the Optum medical claims database in the United States also compared patients taking fluoroquinolones to those prescribed a beta-lactam antibiotic since they may be prescribed for similar conditions; there was no association between uveitis and fluoroquinolone use, but there was an association between systemic illnesses that may be associated with uveitis and fluoroquinolone use (72). These authors proposed that fluoroquinolones may be more likely to be prescribed in patients with a systemic illness that may predispose individuals to develop uveitis rather than directly causing the uveitis.

##### Immune Checkpoint Inhibitors

Immunotherapies are a newer treatment modality for many types of cancers. Immune checkpoint inhibitors represent one of the classes of immunotherapies that are monoclonal antibodies that block inhibitory receptors often activated by tumor cells to evade the immune response; two such targets include programmed death-1 (PD-1) and cytotoxic T lymphocyte antigen-4 (83). Because of the action of these therapies in promoting T cell responses to cancer cells, they also increase the likelihood of autoimmune adverse effects, including uveitis. Several medications, including ipilimumab, nivolumab, and pembrolizumab, have been associated with ocular adverse effects. Using the Food and Drug Administration Adverse Events Reporting system, one study conducted a disproportionality analysis to compare adverse drug reactions associated with immune checkpoint inhibitors with rates reported with all other drugs (83). Ipilimumab and nivolimumab had the strongest associations with uveitis (reporting odds ratio: 10.53, 95% CI 7.30–15.22 and 8.73, 95% CI 6.25–12.20, respectively), whereas pembrolizumab had a higher association with ocular myasthenia (83). Unlike epidemiologic studies using cohort or case-control designs, disproportionality analysis does not account or adjust for other factors such as co-morbidities that may predispose to autoimmune disease or patient demographic information.

From a recent large retrospective cohort study using insurance claims database, Xia et al. showed that patients exposed to checkpoint inhibitors had a 0.3% incidence of non-infectious uveitis, which was significantly higher compared to patients on non-checkpoint inhibitor chemotherapy (73). Similarly, Fortes at el. found a 0.4% incidence of uveitis in patients exposed to checkpoint inhibitors, although they did not specifically identify uveitis as non-infectious (74).

##### Hormone Replacement Therapy

As previously discussed, female sex hormones play a role in the development of autoimmune disease. In a recent study, Sobrin et al. found that patients on female hormone therapy (FHT), which includes menopausal hormonal replacement and hormone contraceptive therapy, had ~20% increased chance of developing non-infectious uveitis. This study also showed that this association was highest for anterior uveitis (HR 1.23, 95% CI, 1.05–1.45) and for women > 44 years of age (HR 1.23, 95% CI, 1.03–1.47). The trend was similar in women <44 years old, but not statistically significant. Overall, the absolute risk of non-infectious uveitis with FHT was fairly low, suggesting that it is a safe therapy for most patients (75).

##### Etanercept

Anti-Tumor necrosis factor alpha (Anti-TNF-alpha) agents are increasingly used in the treatment of immune-mediated disorders. Etanercept (Enbrel) is an anti- TNF-alpha agent which also has activity against TNF-beta, and is commonly used in the treatment of rheumatoid arthritis, psoriatic arthritis, and ankylosing spondylitis. Several reports have identified cases of non-infectious uveitis after initiation of etanercept therapy (76, 84). In a study by Lim et al. the authors reviewed two large databases for cases of uveitis reported in the US associated with etanercept, infliximab, and adalimumab therapy. After adjusting for underlying disease, age and sex, they found that the number of uveitis cases associated with etanercept (20 cases) exceeded that of infliximab (4 cases) and adalimumab (2 cases) (*p* < 0.001). In four cases of uveitis associated with etanercept, the inflammation resolved with cessation of etanercept and recurred upon re-challenging in two of the patients. The study however is limited by incomplete clinical information available in the databases (i.e., underlying disease unknown in 50% of cases). Thus, is it possible that some of the included cases of uveitis associated with anti-TNF-alpha inhibitors, are actually being treated with the drugs (85). In a meta-analysis of uveitis rates associated with etanercept for ankylosing spondylitis across 8 clinical trials, Sieper et al. found that uveitis rates were lower for etanercept than for placebo, in placebo-controlled trials. This study was limited in that prior history of uveitis was not specifically reported (77). In a recent retrospective study from France, the risk of anterior uveitis occurrence did not appear to differ in patients with spondyloarthropathies treated with etanercept and monoclonal antibodies (infliximab and adalimumab). This study was also limited by single center nature, monitoring bias, and recall bias (78).

## Conclusions and Future Directions

Non-infectious uveitis is a leading cause of visual impairment and blindness worldwide. In this systematic review we summarized the prevalence and incidence of uveitis, and described the modifiable and non-modifiable risk factors specific for non-infectious uveitis in adults over the last 50 years. Understanding the epidemiology of non-infectious uveitis can help clarify the burden of disease and populations at risk, which in turn can help with resource allocation for managing chronic disease. Non-infectious uveitis can be a particularly difficult diagnosis for both patients and physicians. Patients struggle to understand why there is an immunologic process affecting their vision, and physicians often cannot find a “cause” for the immunologic process. The aim of this systematic review is to help clinicians identify potential disease contributing factors for patients with non-infectious uveitis, which is important in diagnosis and management of disease.

Several risk factors have been identified as potentially contributing to the development of uveitis, including tobacco exposure, vitamin D deficiency, and pre-eclampsia/eclampsia. Pregnancy has been found to be protective for uveitis flares. Systemic medical conditions including diabetes, thyroid disease and celiac disease have been found to be associated with an increased risk of non-infectious uveitis. Also, certain medications such as bisphosphonates, fluoroquinolones, immune checkpoint inhibitors, female hormone replacement therapy, and etanercept have been associated with increased risk of non-infectious uveitis. In the clinical setting, uveitis patients' medications should be thoroughly reviewed to identify any culprit medications that may have increased the risk of non-infectious uveitis. Counseling on smoking cessation should also be part of the discussion in the management of chronic non-infectious uveitis.

Future studies in uveitis will include more large-scale, multi-center studies that focus on risk factor and lifestyle modifications in the prevention and management of non-infectious uveitis. Analysis of big data, including the Intelligent Research in Sight (IRIS) registry developed through the American Academy of Ophthalmology as the nation's first comprehensive eye disease clinical registry, may allow for innovative ways to perform epidemiologic studies and outcomes-based studies on patients with uveitis. Understanding populations at risk and modifiable factors that may be involved in the development of uveitis can help in disease prevention, monitoring and control to decrease overall morbidity from this potentially blinding condition.

## Data Availability Statement

The original contributions presented in the study are included in the article/supplementary material, further inquiries can be directed to the corresponding author/s.

## Author Contributions

KJ and A-ML-C contributed to the study design, literature review, data analysis, and writing of the manuscript. A-ML-C received funding for support of this manuscript. All authors contributed to the article and approved the submitted version.

## Funding

This work was supported by grants: P30 EY001792, NIH/NEI K12 EY021475, UIC CCTS 2019-01, unrestricted departmental funding from Research to Prevent Blindness.

## Conflict of Interest

The authors declare that the research was conducted in the absence of any commercial or financial relationships that could be construed as a potential conflict of interest.

## Publisher's Note

All claims expressed in this article are solely those of the authors and do not necessarily represent those of their affiliated organizations, or those of the publisher, the editors and the reviewers. Any product that may be evaluated in this article, or claim that may be made by its manufacturer, is not guaranteed or endorsed by the publisher.
